# Linking Primary and Secondary Care after Psychiatric Hospitalization: Comparison between Transitional Case Management Setting and Routine Care for Common Mental Disorders

**DOI:** 10.3389/fpsyt.2016.00096

**Published:** 2016-06-02

**Authors:** Charles Bonsack, Philippe Golay, Silvia Gibellini Manetti, Sophia Gebel, Pascale Ferrari, Christine Besse, Jérome Favrod, Stéphane Morandi

**Affiliations:** ^1^Community Psychiatry Service, Department of Psychiatry, Consultations de Chauderon, Lausanne University Hospital (CHUV), Lausanne, Switzerland; ^2^La Source School of Nursing Sciences (HEdS La Source), University of Applied Sciences Western Switzerland, Lausanne, Switzerland

**Keywords:** case management, discharge planning, mental health care, psychiatry, readmission

## Abstract

**Objectives:**

To improve engagement with care and prevent psychiatric readmission, a transitional case management intervention has been established to link with primary and secondary care. The intervention begins during hospitalization and ends 1 month after discharge. The goal of this study was to assess the effectiveness of this short intervention in terms of the level of engagement with outpatient care and the rate of readmissions during 1 year after discharge.

**Methods:**

Individuals hospitalized with common mental disorders were randomly assigned to be discharged to routine follow-up by private psychiatrists or general practitioners with (*n* = 51) or without (*n* = 51) the addition of a transitional case management intervention. Main outcome measures were number of contacts with outpatient care and rate of readmission during 12 months after discharge.

**Results:**

Transitional case management patients reported more contacts with care service in the period between 1 and 3 months after discharge (*p* = 0.004). Later after discharge (3–12 months), no significant differences of number of contacts remained. The transitional case management intervention had no statistically significant beneficial impact on the rate of readmission (hazard ratio = 0.585, *p* = 0.114).

**Conclusion:**

The focus on follow-up after discharge during hospitalization leads to an increased short-term rate of engagement with ambulatory care despite no differences between the two groups after 3 months of follow-up. This short transitional intervention did, however, not significantly reduce the rate of readmissions during the first year following discharge.

**Trial registration number:** ClinicalTrials.gov Identifier NCT02258737.

## Introduction

The movement of deinstitutionalization transformed care provision in most Western Countries during last decades (1, 2). The number of psychiatric admissions has increased, whereas the number of psychiatric beds has decreased (3). Mental health teams are now faced with an increased number of discharges and have less time to prepare them. Aftercare provision is one of the most consistent predictors of rehospitalization (4) and attendance at outpatient appointments after discharge reduces early readmissions (5). In a recent study, among individuals who had been discharged from a hospital closest to their death by suicide, three-quarter died in the month following discharge, and the most consistent modifiable factor associated with death in the month following last contact was number of outpatient consultations following discharge (6). In this context, linking with primary and secondary care after psychiatric hospitalization is a particular challenge.

A literature review by Steffen and colleagues has shown that discharge planning intervention improved adherence to after care and reduced readmissions among people with a severe mental illness (7). The authors mentioned that most of the studies were conducted in the USA, Canada, and the UK and the findings were not generalized in other countries. Another concern was the heterogeneity of diagnosis and a broad variation in post-discharge problems. In another literature review, Vigod and colleagues reported, however, that only 7 out of 15 studies found a significant reduction of rehospitalizations (8). Previous studies demonstrated that around 50% of hospitalized psychiatric patients did not attend their scheduled or rescheduled outpatient appointment after discharge (9, 10). A pilot study showed that primary and secondary care hospitalized patients tend to have a less severe illness and a better social functioning than heavy users of acute psychiatric care but that their distress and needs tended to be underestimated during hospitalization (11). Moreover, their profile of mid age women with personality and mood disorder correspond to those patients most at risk of suicide during the weeks following discharge of psychiatric hospitalization (12). To improve the focus on establishing follow-up after discharge, a “transitional case management” intervention has been developed. This is a short, structured intervention which follows the same principles as critical time intervention (13). It is started during the hospitalization and continues for 1-month after discharge. The intervention is aimed at patients who return home after discharge and who are followed up by a general practitioner or a private psychiatrist. It aims to improve engagement with ambulatory care and reduce the risk of relapse and readmission (11).

### Aim of the Study

This study tests whether transitional case management improves engagement with ambulatory care 1 year after psychiatric hospitalization and whether the intervention affects readmission rate during the year following discharge compared to routine treatment. The first outcome was defined as whether transitional case management intervention improved engagement with care, measured as number of contact with ambulatory care, during the follow-up after discharge. The second outcome was defined as whether transitional case management intervention had an impact on the rate of readmission during the 12 months following discharge.

## Materials and Methods

### Participants

This study is a randomized controlled trial (ClinicalTrials.gov Identifier NCT02258737). Eligible patients were those hospitalized in the admission ward of the psychiatric hospital of Cery in Lausanne, returning home after discharge and followed up by a general practitioner or a private psychiatrist (primary or secondary outpatient care). They were aged between 18 and 65 years. Patients suffering an organic disorder or non-French speaking subjects and those followed up within the university psychiatric services were excluded (tertiary outpatient care). The study was approved by the Biology and Medicine faculty Ethics Committee of Lausanne University. Patients were informed about the confidentiality of data and their right to withdraw from participation at any time. Written informed consent was obtained from all patients.

Immediately after initial assessment, each patient was randomized and assigned to either treatment as usual or to transitional case management. Randomization was in blocks of eight, based on a computer-generated allocation placed in closed envelopes. Envelopes were generated and kept by a member of the administrative staff of the project. Initial and follow-up assessments were conducted by six research psychologists who had been trained prior to the study to ensure inter-rater reliability.

### Procedures

#### Treatment as Usual

Patients allocated to treatment as usual were referred to a general practitioner or a private psychiatrist after discharge.

#### Transitional Case Management Intervention

In the transitional case management group, a case manager, a nurse, or a social worker was added to the treatment as usual procedure. Their role was not to replace the other care providers but to coordinate care provision and to represent the patient’s viewpoint. Transitional case management followed the same nine target areas as critical time interventions to improve continuity of care: system coordination, engagement in psychiatric care, continuation of substance abuse treatment, medication adherence, family involvement and social support network, life skills training and support, integration of medical care, establishment of community linkage, and practical needs assistance (13). Intervention was structured in six steps (14). First, every patient who was to be followed by primary or secondary care was identified at admission. Second, a first contact with the patient was made during hospitalization to propose intervention and evaluate the demands. Third, an evaluation was done with two or three appointments, some of them with the patient alone, other with members of the patient’s network, using specific clinical tools:

(1) “Echelle lausannoise d’autoévaluation des besoins” (ELADEB), a self-administrated scale that determines patient’s needs and expectations through visual cards classified by the patient (15).
(2) “Carte réseau,” a self-representation of the personal network through which the patient identified people, professional or not, that could provide help after discharge.
(3) A “Joint crisis plan” constructed with the case manager (16).

Since discharge, most contacts took place in the community outside the office, up to twice a day if necessary. The fourth step was a home visit, which insured that the discharge plan was realistic and that the network was available. Joint crisis plan was readjusted if necessary. Fifth, during the month after discharge, the transitional case management is adapted according to the needs of patients: minimal was phone calls and being available on demand, standard was four contacts during the follow-up, intensive was more than four contacts with home visits up to twice a day. The case manager often attended appointments (e.g., medical, social work, welfare) with the patient. Sixth, the intervention ended with a meeting between the patient, the transitional case manager, and the medical doctor in charge. A written report was delivered.

### Measures

Data on contact with ambulatory care and social functioning were provided by interviews during follow-up assessments (after 1, 3, 6, and 12 months). Social functioning was assessed using the Global Assessment of Functioning (GAF) (17) and clinical status at baseline using the symptom check-list (SCL-90R) global score (18, 19). Data on readmissions were provided by hospital records.

### Analysis

The first outcome was defined as whether transitional case management intervention improved engagement with care during the follow-up after discharge. The dependant variable was the number of contact with ambulatory care between 0 and 1, 1 and 3, 3 and 6, and 6 and 12 months after discharge. Because of the count nature of the dependant variable, the comparison between groups was performed using a Poisson regression model. The potential influence of age, sex, level of education, initial level of social functioning, and familial situation was controlled for in an adjusted model. Only significant covariates were included in this additional model. Power calculations for the Poisson regression were based on estimated number of contact with ambulatory care. Given a base rate of 2 contacts with ambulatory care in the treatment as usual group, we could test a 50% increase of the number of contacts with a power of 0.80 with 48 patients per group.

The second outcome was defined as whether transitional case management intervention had an impact on the rate of readmission during the 12 months following discharge. The dependant variable was the duration before first psychiatric readmission. A continuous-time survival analysis was performed using the Cox regression model. The potential influence of age, sex, level of education, initial level of social functioning, and familial situation was controlled for in an adjusted model. Only significant covariates were included in this additional model. We anticipated an event rate of 0.4 and a SD of 0.5 for the group covariate, which would allow us to detect a hazard ratio of 1/3 with a power of 0.80 with 33 observations per group.

Comparisons in terms of demographic and baseline characteristics between the two groups were performed with independent *t*-tests for continuous variables. For categorical variables, analyses were performed using Pearson’s Chi-Square tests. All statistical tests were two-tailed and significance was determined at the 0.05 level. All statistical analyses were performed with the Mplus statistical package version 7.4 and IBM SPSS version 22.

## Results

**Figure 1:**
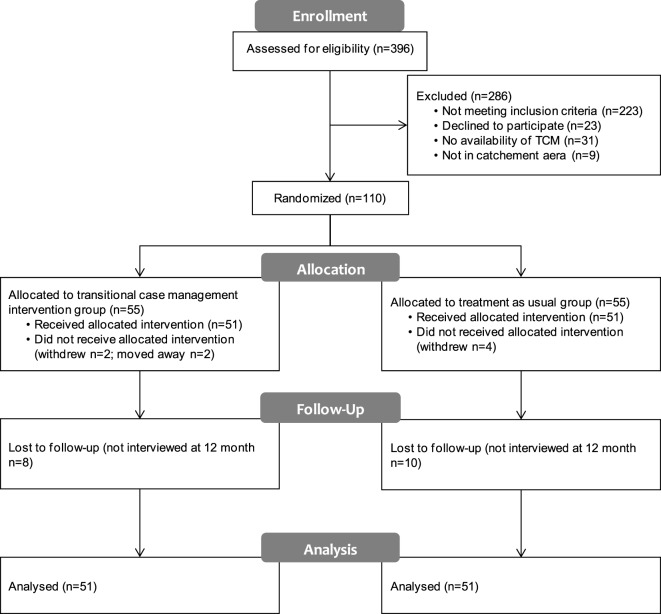
**Participant flow**.

Figure 1 summarizes the participant flow. On 396 patients admitted to the “Admission, Orientation, Crise” service during the 17 months of recruitment, 223 (56.3%) were ineligible as they were followed up by the university clinics, were not aged between 18 and 65 years, presented an organic disorder or had significant difficulties in understanding French. One hundred seventy-three (43.7%) fulfilled inclusion criteria and were discharged to follow-up by a general practitioner or a private psychiatrist. Although eligible, 23 (13.29%) patients refused to participate to the study. The transitional case management team was not able to provide an intervention for 40 (23.12%) people: the admission time was too short for 24 patients, the case manager had no availability for 7 patients, and 9 did not live in a catchment area. One hundred ten patients completed the consent form and were randomized. Eight patients, four in each arm, did not attend the baseline interview after randomization. Two moved away in the intervention group and six others withdrew. One hundred two patients were randomly allocated to discharge with transitional case management intervention (*n* = 51) or with a treatment as usual (*n* = 51). Eighty-four (82.4%) patients were interviewed after 12 months of follow-up: research psychologists were not able to contact 8 patients in the transitional case management group and 10 in treatment as usual group. Data from the 51 patients from the transitional case management intervention arm and the 51 patients from the treatment as usual arm were analyzed in an intent to treat analysis. There were no differences between the two groups regarding patients’ baseline and clinical characteristics (Table 1). Given the low rate of psychotic patients, this sample could be referred as patients suffering from common mental health disorders.

**Table 1:** **Patient characteristics at baseline**.

Characteristics	Transitional case management group (*n* = 51)	Treatment as usual group (*n* = 51)	Statistic	*p*-Value
**Demographics**				
Age (years)	40.0 (11.9)	41.3 (10.6)	*t*(100) = −0.555	0.580
Sex, % female (*N*)	66.7 (34)	52.9 (27)	χ^2^(1) = 1.998	0.157
**Education**				
Low^a^	31.4 (16)	31.4 (16)		
Intermediate^b^	39.2 (20)	41.2 (21)	χ^2^(2) = 0.059	0.971
High^c^	29.4 (15)	27.5 (14)		
**Familial situation**				
Single	35.3 (18)	29.4 (15)		
Married	29.4 (15)	43.1 (22)	χ^2^(2) = 2.097	0.350
Other^d^	35.3 (18)	27.5 (14)		
**Ethnicity**				
Caucasian	84.3 (43)	92.2 (47)	χ^2^(1) = 1.511	0.219
**Origin**				
Swiss	62.7 (32)	52.9 (27)	χ^2^(1) = 1.005	0.316
**Language**				
Mother tongue French	78.4 (40)	70.6 (36)	χ^2^(1) = 0.826	0.363
**Clinical status**				
Global assessment of functioning	45.5 (5.9)	46.0 (7.0)	*t*(100) = −0.426	0.671
Symptom check-list global score (SCL-90R)^e^	1.1 (0.5)	1.2 (0.7)	*t*(100) = −0.901	0.370
**Duration of illness**				
Less than a year	35.3 (18)	33.3 (17)		
Between 1 and 5 years	33.3 (17)	27.5 (14)	χ^2^(2) = 0.763	0.683
More than 5 years	31.4 (16)	39.2 (20)		
**Clinical history**				
First psychiatric admission	84.3 (43)	76.5 (39)	χ^2^(1) = 0.955	0.318
**Main disorder**				
Affective disorder	52.9 (27)	70.6 (36)	χ^2^(1) = 3.363	0.067
Neurotic, stress-related or somatoform disorder	19.6 (10)	7.8 (4)	χ^2^(1) = 2.981	0.084
Personality disorder	11.8 (6)	5.9 (3)	χ^2^(1) = 1.097	0.295
Psychotic disorder	7.8 (4)	7.8 (4)	χ^2^(1) = 0.000	1.000
Substance use	7.8 (4)	7.8 (4)	χ^2^(1) = 0.000	1.000

*^a^No post school training*.

*^b^Post school training*.

*^c^College/University*.

*^d^Divorced/widowed/separated*.

*^e^Global Severity Index*.

Concerning the first outcome, results of the Poisson regression models at 1, 3, 6, and 12 months are presented in Table 2. During the first month after discharge, the number of contact with ambulatory care was not significantly different between the two groups (*B* = 0.098, *p* = 0.372). Between 1 and 3 months after discharge, transitional case management patients reported more contacts with care service (*B* = 0.371, *p* = 0.004). The mean count of contact in the transitional case management group was 2.79 (SD = 1.42), while only 1.93 (SD = 1.29) in the treatment as usual group (Cohen’s *d* = 0.64; medium effect). Interestingly, the ratio of patients who reported at least one contact with ambulatory care during the same period was high in both groups (100 versus 87.5% in the control group). In the next 3 months of follow-up (3–6 months after discharge), no significant differences of number of contacts remained (*B* = 0.076, *p* = 0.603). Age favorably influenced the contact count (*B* = 0.015, *p* = 0.016) while male patients tended to report a greater number of contacts with ambulatory care (*B* = 0.392, *p* = 0.005).

**Table 2:** **Poisson regression models for the number of contact with ambulatory care**.

	*B*	95% CI	*p*-Value
**0–1 months after discharge**			
Bivariate model			
Intervention	0.098	−0.110 to 0.307	0.372
Intercept	0.907	0.728–1.086	<0.001
**1–3 months after discharge**			
Bivariate model			
Intervention	0.371	0.117–0.626	0.004
Intercept	0.655	0.450–0.860	<0.001
**3–6 months after discharge**			
Bivariate model			
Intervention	0.076	−0.210 to 0.362	0.603
Intercept	0.808	0.613–1.003	<0.001
Adjusted model			
Intervention	0.071	−0.189 to 0.331	0.593
Age	0.015	0.003–0.028	0.016
Sex	0.392	0.116–0.668	0.005
Intercept	−0.108	−0.741 to 0.525	0.739
**6–12 months after discharge**			
Bivariate model			
Intervention	0.108	−0.147 to 0.363	0.406
Intercept	0.840	0.644–1.037	<0.001
Adjusted model			
Intervention	0.097	−0.134 to 0.329	0.410
Age	0.010	0.001–0.020	0.035
Sex	0.423	0.183–0.664	0.001
Intercept	0.130	−0.334 to 0.594	0.740

*CI, confidence interval*.

Finally, during 6–12 months after discharge, no difference in the number of contact could be observed (*B* = 0.108, *p* = 0.406). However, the positive effect of male gender (*B* = 0.423, *p* = 0.001) and greater age (*B* = 0.010, *p* = 0.035) could still be observed. Overall, the intervention leaded to a moderately increased short-term rate of engagement with ambulatory care despite no differences between the two groups after 3 months of follow-up. In contrast to age and gender, the general level of social functioning at baseline and education were not related to the number of contacts with ambulatory care.

**Figure 2:**
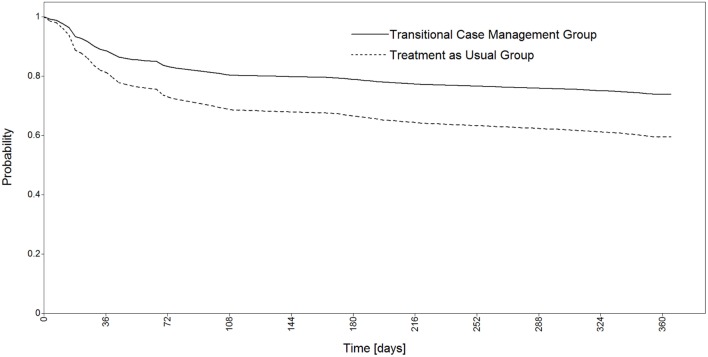
**Cox regression survival curves for the duration before first psychiatric readmission**.

Results of the continuous-time survival analysis are reported in Table 3. The Cox regression model revealed no statistically significant beneficial impact of the transitional case management intervention on the rate of readmission (hazard ratio = 0.585, *p* = 0.114; Figure 2). The rate of readmission in the treatment as usual group (43.1%) was similar as those observed in the same hospital during the two previous years (respectively, 44.7 and 45.5%), whereas rate of readmission in the transitional case management group was 27.5% although this difference failed to reach statistical significance. A high level of education proved, however, to be a preventing factor against readmission (hazard ratio = 0.292, *p* = 0.011). It should also be noted that the general level of social functioning at baseline was not related to the probability of readmission.

**Table 3:** **Cox continuous-time survival analysis of the duration before first psychiatric readmission**.

	*B*	Hazard ratio	95% CI hazard ratio	*p*-Value
**Bivariate model**				
Intervention	−0.537	0.585	0.301–1.137	0.114
**Adjusted model**				
Intervention	−0.540	0.583	0.300–1.132	0.111
Education (high)	−1.232	0.292	0.113–0.752	0.011
Education (low)	−0.429	0.651	0.350–1.362	0.255

*CI, confidence interval*.

## Discussion

Transitional case management leaded to a moderately increased short-term rate of engagement with ambulatory care despite no differences between the two groups after 3 months of follow-up. This may indicate that it is the focus on preparing for engagement, rather than the specifics of the transitional case management process that is particularly effective. Globally, the rate of engagement with care was, however, much higher than it was in the only previous study carried out in Lausanne which mostly included younger male patients with first episode psychosis (10). This rate was also considerably above the average rate of 50% identified in the wider literature for follow-up after acute hospitalization (9). These results suggest that linking with primary and secondary outpatient care is better for these patients than linking with tertiary care for more severe and persistent illness.

This short transitional intervention did, however, not reduce significantly the rate of readmissions during the first year following discharge. The rate of readmission in the transitional case management group was not significantly lower than in the treatment as usual group or those generally observed in the same hospital. This lack of important decrease may suggest that case management does not markedly reduces the rate of readmission during the year following discharge and is in accordance with the results of a systematic review about the effectiveness of transitional interventions to reduce psychiatric readmissions in adults (8). Three other studies recently tested a similar intervention and did not find a reduction in either rehospitalization rates (13, 20, 21). The Hengartner et al. study (21) focused on low-frequency users which could be comparable to our sample. The Puschner study included more highly impaired high-frequency users (20), whereas the Dixon study sample could be considered as “intermediate” (13).

Age and male gender showed to increase the number of contact with ambulatory care. A high level of education also showed to be a preventing factor against readmission. Finally, it should be noted that the general level of social functioning at baseline was neither related to the number of contact with ambulatory care nor the probability of psychiatric readmission. These findings may be explained by the focus on a population of higher functioning independent mid age patients who need more support to prevent losses (job, couple, housing) linked to the psychiatric episode.

In fact, most of the research concerning transitional interventions has focused on “revolving door” patients or on severe mentally ill patients suffering a psychosis or a bipolar disorder (22, 23). In this study, the profile of the population differed. The majority of the patients were women. They were married and employed at the moment of their baseline hospitalization. The main diagnosis was an affective, neurotic, stress related, or somatoform disorder in most of the situations. Few patients suffered from psychotic disorder. The duration of the illness was more than 1 year for two-thirds of the patients, but the baseline hospitalization was the first one for the majority of them. The transitional case management concerns itself with specific patients who go through a life crisis and may potentially lose their social situation. These patients may be neglected during their hospitalization, when ward teams are busy with more severe cases. This population is also at high risk to commit suicide in the first weeks after a psychiatric hospital discharge (12).

### Potential Shortcomings and Limitations

Limitations of this study are low sample size and unique site implementation: replication is therefore needed. The results also relied on a small subsample of all patients initially screened for eligibility. Generalizability of the results may thus be restricted.

## Conclusion

This 1 month transitional intervention produced a moderately increased short-term rate of engagement with ambulatory care, but no significant reduction in the rate of readmissions during the first year following discharge. Its conception and effectiveness were comparable to the 9 months critical time intervention (24), while focusing on less severe common psychiatric disorders that link with primary or secondary outpatient care after discharge. This suggests that several forms of transitional case management may be necessary to meet the different needs of hospitalized psychiatric patients. Considering deinstitutionalization in psychiatry, more research is needed to study and improve the link between tertiary and primary care.

## Author Contributions

CB, SGM, PF, and JF contributed to the conception and design of the study. SGM, SG, PF, CB, and SM contributed to the acquisition of the data. PG, SGM, SG, SM, CB, and CBe contributed to data analysis and interpretation of the data. SM and CB drafted the manuscript. PG, SGM, SG, PF, CBe, and JF were involved in the critical revision of the manuscript. All authors have given final approval of the version to be published.

## Conflict of Interest Statement

The authors declare that the research was conducted in the absence of any commercial or financial relationships that could be construed as a potential conflict of interest.
